# The Development of a New Location-Based Accessibility Measure Based on GPS Data

**DOI:** 10.3390/s25206274

**Published:** 2025-10-10

**Authors:** Feng Liu, Ansar Yasar, Jianxun Cui, Davy Janssens, Geert Wets, Mario Cools

**Affiliations:** 1 Transportation Research Institute (IMOB), Hasselt University, 3500 Hasselt, Belgium; ansar.yasar@uhasselt.be (A.Y.); davy.janssens@uhasselt.be (D.J.); geert.wets@uhasselt.be (G.W.); 2 School of Transportation Science and Engineering, Harbin Institute of Technology, Harbin 150001, China; cuijianxun@hit.edu.cn; 3 Chongqing Research Institute of HIT, Chongqing 401100, China; 4 LEMA, University of Liège, 4000 Liège, Belgium; mario.cools@uliege.be

**Keywords:** location-based accessibility, impedance function, travel time distribution, GPS data

## Abstract

Accessibility is a key dimension for sustainable transport network management and planning. However, conventional location-based accessibility measures typically rely on average travel times as the sole temporal metric, neglecting detailed travel time distributions. Consequently, these methods yield identical accessibility values for study zones with the same mean travel time but different travel time variations. To overcome this limitation, we developed a novel approach that explicitly integrates the probability density distributions of travel times, modelling the impact of travel time variability on accessibility. We applied the proposed method using GPS data collected from taxis in Harbin, China, and compared its outcomes with those from existing potential accessibility calculations. Across all 103 study zones in Harbin, the existing method underestimated the accessibility by 6–28%, with an average underestimation of 17% when benchmarked against the new method. These inaccuracies also impaired the identification of urban areas with the lowest accessibility levels, leading to the misclassification of 20% of problematic zones. The findings highlight the limitations of existing methods, which produce biassed accessibility estimations and misleading results. In contrast, the proposed travel time variability-integrated accessibility measure demonstrates greater sensitivity to actual traffic conditions, providing a more accurate and objective assessment of network performance.

## 1. Introduction

### 1.1. Problem Statement

With the continuing urbanisation of the world’s population and economic growth of cities, spatial urban areas are expanding, and new communities and activity locations are becoming decentralised. However, existing transport networks have not developed at the same pace as urban growth, generating isolated pockets of areas that are difficult to reach by the transport systems [1]. It is thus important to conduct a timely examination of the changing land-use structure and travel conditions and accurately evaluate accessibility. This helps identify poorly reachable areas and informs efforts to improve the accessibility of these places as well as the accessibility of the city as a whole [2].

Location-based potential measures have been widely applied to analyse the accessibility of an urban area [3,4,5]. These measures use an impedance function, typically the negative exponential function (*NEF*) (i.e., *f_t_* = *e^−kt^*, where *t* is the travel time and *k* is a controlling parameter), to characterise the declining attractiveness of activities in a zone to a destination as the average travel time between these two places increases, in order to take into account the effects of travel times on people’s perceptions of activity attractiveness.

Nevertheless, the potential measures only consider a single time point (i.e., the average travel time) while neglecting detailed travel time distributions, resulting in the same measure to an activity place for two study zones that share identical mean travel times but differ in travel time distributions (e.g., the standard deviations). According to *NEF*, for individual trips, the number of changes in the attractiveness of activities resulting from the changes in travel times is non-linearly distributed; the longer the time, the more diminishing the attractiveness and the lower the accessibility. This non-linear relationship implies that the actual value of accessibility is more decided by trips with shorter travel times than those featuring longer times. The zones displaying varied travel time distributions should thus be assigned different measures. For zones with smaller deviations, the distribution of travel times is more centred around the mean travel time, generating fewer trips with shorter times, leading to a relatively higher level of diminishing activity attractiveness and, consequently, a lower level of accessibility. In contrast, regarding zones with larger deviations, travel times are more dispersed, causing more trips with shorter times, resulting in a lower level of reduction in attractiveness and, consequently, a higher level of accessibility (see further elaboration in the Section The New Measure).

In order to characterise the differences in accessibility for varied travel time distributions, in this study, we extended the potential measure to incorporate travel time distributions into the impedance function based on GPS data of urban vehicles. Compared with existing *location-based measures* (LBMs), the new method offers several key advantages:

1. It constructs a new impedance function by integrating the probability distribution of travel times, considering both the mean and variation in individual travel times. By combining travel time distributions, the new measure accommodates the effect of each individual trip, whereas existing measures only consider the effect of the mean travel time.
2. Given that traffic conditions in an urban road network are highly divergent and travel times are stochastic (even within the same period of the day), the proposed method captures the statistical fluctuations of travel times and provides a more accurate and realistic assessment of network accessibility.
3. In many cities worldwide, GPS devices are installed in taxis and in many other urban vehicles—such as private cars, buses and trucks, generating massive GPS data and enabling the extraction of travel time distributions. This makes the approach cost-effective, timely-updated and easily transferrable to other cities.

The remainder of this paper is organised as follows. Section 1.2 describes the state-of-the-art accessibility research, while Section 2 introduces the data and explains the proposed method. Section 3 presents a case study, and Section 4 ends this paper with major discussions and conclusions.

### 1.2. State-of-the-Art Accessibility Research

#### 1.2.1. Accessibility

Accessibility is defined as the ease and extent to which land-use and transport systems enable individuals to reach activities and destinations using certain transport modes, e.g., the number of jobs accessible within 30 min by car [6]. It not only considers travel conditions (e.g., travel times or distances) but also the distribution of land-use and activity locations across the transport network [7].

Various methods have been developed for measuring accessibility, including infrastructure-based [6], person-based [8], location-based [9], and utility-based [10] approaches. Particularly, location-based measures (LBMs) analyse accessibility from a locational perspective, and are mostly applied owning to their good operability and interpretation [11]. Three major categories of LBMs exist, consisting of contour (cumulative opportunity), potential (gravity), and 2-step floating catchment area (2SFCA) measures. A contour measure represents the total number of activities that can be reached from the study zone within a predefined travel time threshold [4]. In contrast, a potential measure uses an impedance function to reduce the attractiveness of activities to the study zone, as the travel time between the study and activity zones increases [5]. Furthermore, 2SFCA incorporates the competitive relationship between activity supply and demand, accounting for the fact that access to opportunities is affected not only by geographical proximity and travel times, but also by the competition of many people trying to access the same opportunity [12,13].

Traditionally, all the above measures are derived through a static analysis; travel time is calculated as the average travel time per day, obtained from travel surveys or static sensors, while the attractiveness of activities is quantified as the total number or size of activities within activity zones [6,14]. Consequently, the resulting measures remain fixed throughout the day and fail to capture the dynamic nature of accessibility, which fluctuates across different time periods due to variations in human activity patterns, traffic conditions and activity availability (e.g., shop opening and closing hours). This lack of temporal differentiation renders the measures inadequate for reflecting actual accessibility at varied times of the day.

#### 1.2.2. Dynamic Accessibility

With advancements in information and communication technologies, it has become technically and economically feasible to collect and process large amounts of mobility data (e.g., GPS, mobile phone and social media data) and activity data (e.g., crowdsourced information on business establishment) [15]. These data facilitate the extraction of detailed insights into human activity patterns, service demand and traffic conditions, presenting great opportunities to refine conventional accessibility measures. Based on such data, time-dependent *dynamic location-based measures* (D-LBMs) have been developed to capture temporal variations in accessibility across different time periods of the day [3,5]. For example, Järv et al. [16] incorporated the temporal dynamics of activities and travel—such as grocery store opening hours, time-dependent traffic conditions and travel times—to compute hourly food accessibility. By comparing these hourly measures with conventional static analyses (e.g., using average daily travel times), they found that static measures typically overestimate people’s access to potential opportunities. Similarly, Hu and Downs [17] presented a framework for measuring and visualising hourly job accessibility, accounting for temporal fluctuations in job supply, worker demand and travel times. Furthermore, Cuervo et al. [18] classified traffic congestion into nine levels, from free-flow (level 1) to peak traffic (level 9), and examined accessibility to medical services at each level. Their results revealed substantial variations; accessibility during peak traffic was 53% lower than under free-flow conditions.

Nevertheless, although D-LBMs account for inter-period variability (e.g., each hour or each congestion level), they still assume that travel times within each period are deterministic, and use the average travel time for accessibility calculation. However, given the stochastic nature of travel demand and traffic conditions in urban road networks, travel times even within the same period are not consistent but subject to variability [19,20]. Travel time variability has been particularly addressed in *reliability-based location-based measures* (R-LBMs), which integrate travel time reliability—the probability of reaching an activity location within a specified time budget—into accessibility analysis [21]. The rationality is that, variability, as a form of travel-time uncertainty, affects how people perceives facilities and therefore should be included in accessibility studies [22]. However, despite the integration of variability, R-LBMs basically still rely on a single time point (e.g., a specific percentile of the travel time distribution) for accessibility estimations, without fully considering the entire distribution of travel times across the study period.

#### 1.2.3. Limitations of Current Accessibility Measures

Both D-LBMs and R-LBMs have proven their value and feasibility in capturing temporal variations, providing deeper insights and more advanced approaches for accessibility analysis [23,24,25]. Nevertheless, these measures compute accessibility using either the mean or a specific percentile of travel time distributions between study and activity zones, assuming that all the travellers experience the same travel time during the analysed period. Neither measure considers the detailed travel time distribution. According to literature [4,6,9], different travel times influence activity attractiveness to varied degrees. For instance, based on NEF, a negative non-linear relationship exists; the longer the (average) travel time, the more the activity attractiveness and accessibility diminish. Thus, accessibility should be differentiated not only across locations and time periods but also among individuals’ trips and perceptions shaped by the varied travel times. Researchers have indeed questioned the underlying assumption of LBMs that all individuals at a given location have equal awareness of activity destinations [26,27,28]. This assumption overlooks the diverse experiences and perceptions of individual travellers. Existing measures, particularly D-LBMs, strive to overcome this weakness by splitting a day into short periods and use average travel times for each period. However, they still assume homogeneous travel times and accessibility within each period and therefore only partially resolve the problem. Consequently, a method that models individual trips and explicitly accounts for travellers’ heterogeneous experiences remains lacking.

The growing availability of GPS data provides accurate routes and travel times for many people, offering detailed spatial and temporal information and near real-time traffic conditions [29]. This makes it possible to analyse accessibility at the level of individual trips. In this study, we incorporate travel time distributions into the calculation of LBMs and derive accessibility based on individual travel times (trips), using taxi GPS data. The key differences between the new and existing measures lie in how they utilise travel times and model the effects of these times. The new measure aggregates the effect of every trip and duration across a period, while existing measures typically analyse the effect of the average duration or another single percentile of the travel time distribution. As a result, the new approach produces accessibility estimates that are more sensitive to individuals’ diverse views and practices, even for the same zone pairs within the same time period.

## 2. Materials and Methods

### 2.1. Data

The GPS data were collected from all licenced taxis in Harbin, the capital of Heilongjiang province in China, totalling 16,000 vehicles. Data were recorded every 30 s during the day and every 2 min at night, generating 1.6 GB of data and 24 million GPS points each day. According to the data, each taxi completes an average of 30 passenger trips per day, resulting in a total of 0.48 m passenger trips. By comparison, Harbin has approximately 1 million private cars, which collectively produce an estimated 2.41 million trips daily, assuming that each car generates an average of 2.41 trips per day [30]. Consequently, taxi passenger trips account for 17% of the total personal travel undertaken in the urban area daily. This highlights the significant role of taxis in meeting urban travel demand for private trips. Further information on the data can be referred to in the paper [30].

For this study, GPS data collected between July and September 2016 were used. The dataset includes variables such as taxi vehicle IDs, GPS coordinates, recording times and status messages indicating whether passengers were on board. A digital map of the road network, obtained from the Baidu Map Open Platform [31], was also utilised. This dataset provides the coordinates and classifications of all activity locations (16,625 in total, spanning 16 types) across the urban area. The classification of activity types is described in Table A1 in Appendix A, and this study performs accessibility analysis regarding the integration of all these types.

### 2.2. Methodology

**Figure 1:**
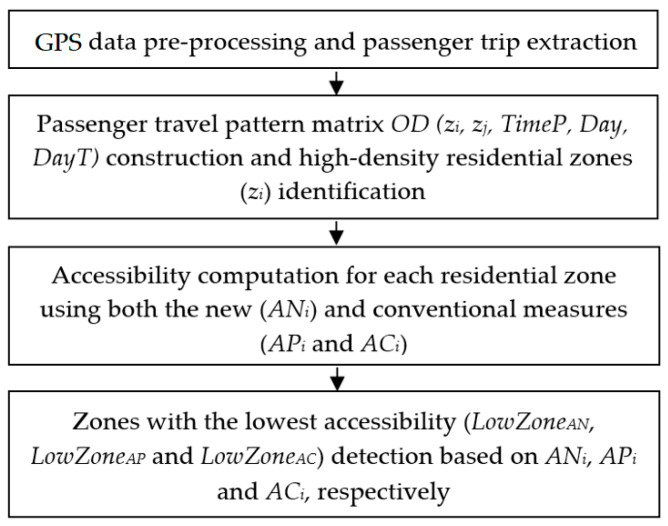
Overall structure of the proposed method. Note: *OD* (*z_i_*, *z_j_*, *TimeP*, *Day*, *DayT*) denotes the passenger travel pattern matrix, where *z_i_* and *z_j_* are the origin and destination zones and *TimeP*, *Day* and *DayT* specify the temporal attributes (time period, calendar day, and day type). *AN_i_*, *AP_i_* and *AC_i_* denote the new, existing potential, and contour measures for study zone *z_i_*, while *LowZone_AP_*, *LowZone_AP_* and *LowZone_AC_* indicate the zones with the lowest *AN_i_*, *AP_i_* and *AC_i_* values, respectively.

The proposed method consists of four main steps (See Figure 1): (i) pre-processing GPS data and extracting passenger trips, (ii) constructing passenger travel patterns and identifying high-density residential zones, (iii) calculating accessibility for each residential zone using both the new and conventional measures, and (iv) identifying zones with the lowest levels of accessibility.

#### 2.2.1. GPS Data Pre-Processing and Passenger Trip Extraction

Let *p*_1_ (*l*_1_, *t*_1_, *s*_1_)-…-*p_n_* (*l_n_*, *t_n_*, *s_n_*) represent a GPS trajectory from a taxi on a day, where each point *p_k_* (*k* = 1, …, *n*) consists of a coordinate set *l_k_* = {*x_k_*, *y_k_*}, a time stamp *t_k_*, and a status message *s_k_*, which equals 1 when the taxi is occupied by passengers and 0 when the vehicle is idle and the driver is searching for clients. Let *ED_k_* (*l_k+_*_1_, *l_k_*) denote the geographic distance between *p_k_* and *p_k+_*_1_, calculated using the Haversine formula [32]. The speed *Speed_k_* at *p_k_* is then computed as

(1) $${Speed}_{k}=\frac{ED(l_{k+1},l_{k})}{t_{k+1}-t_{k}}$$

From the GPS trajectories, points with zero coordinates or *Speed_k_* exceeding the threshold *TH_Speed_* are first deleted. *Passenger trips*, where taxis are occupied by clients, are then extracted based on changes in *s_k_* between consecutive points. Specifically, let *Trip* = *p_o_* (*l_o_*, *t_o_*, *s_o_*)-…-*p_d_* (*l_d_*, *t_d_*, *s_d_*) represent a passenger trip, where *p_o_* and *p_d_* are the first and last points, such that *s_k_* = 1 (*k* = *o*, …, *d*) and *s*_*o*−1_ = *s*_*d*+1_ = 0. This study focuses solely on passenger trips, as taxis with passengers on board are better suited to reflect actual traffic conditions, such as driving speeds and travel times. For each passenger trip, the travel time *t*, travel distance *d* and route directness (circuity) *cir* are computed according to Formula (2).

(2) $$\begin{array}{l} t=t_{d}-t_{o}\\ d=\sum_{i=o}^{d-1}[ED(l_{i},l_{i+1})]\\ cir=\frac{d}{ED(l_{o},l_{d})} \end{array}$$

#### 2.2.2. Travel Pattern Construction and High-Density Residential Zone Identification

The entire urban area is divided into *GridX* × *GridY* disjoint zones using a grid-based method. Each zone is denoted as *z_i_* (*i* = 1,…, *GridX* × *GridY*) or *z*(*ix*, *iy*) (*ix* = 1,…, *GridX*; *iy* = 1,…, *GridY*), and each zone pair from *z_i_* to *z_j_* is referred to as *z_ij_* or *z_i_*- *> z_j_*. The temporal dimension of trips is classified into different time periods (i.e., *TimeP*) within a day (i.e., *Day*) and further distinguished by day type (i.e., *DayT*). Based on this spatial and temporal division, a passenger travel pattern matrix *OD*(*z_i_*, *z_j_*, *TimeP*, *Day*, *DayT*) is constructed, with each matrix element representing all the trips that originate from *z_i_*, end in *z_j_*, and start within *TimeP* on *Day* of type *DayT*.

From the matrix *OD*, the average number of trips per day that either originate in *z_i_* in the morning (*mo_i_*) or end in *z_j_* at night (*md_j_*) is calculated for all days of type *DayT*. Zones with both *mo_i_* and *md_j_* exceeding a threshold *TH_M_* are identified as high-density residential areas and used as the study zones. Simultaneously, all activity locations in the city are assigned to zones based on their geographic positions, with zones containing at least one activity location forming the activity zones.

To ensure accurate passenger travel times, two parameters *TH_r_* and *TH_t_* are defined to filter trips that may involve spatial detours (e.g., due to taxi sharing) or temporal extensions (e.g., when taxis stop for an extended period during a trip). Trips with circuity exceeding *TH_r_* or travel times longer than *TH_t_* are considered to involve potential spatial detours or temporal extensions and are removed from *OD*. The travel times of the remaining trips are then used for subsequent accessibility computation.

#### 2.2.3. Accessibility Computation

Based on the obtained study and activity zones as well as passenger travel times, accessibility is computed using both the new and traditional measures. Table A2 in Appendix A summarises all the major variables used in the computation process.

##### The Traditional Measures

The traditional contour measures *AC_ij_* and *AC_i_* for zone pair *z_ij_* and study zone *z_i_*, and potential measures *AP_ij_* for *z_ij_* and *AP_i_* for *z_i_* are computed according to Formula (3) [6] and Formula (4) [9], respectively.

(3) $$\begin{array}{l} h_{ij}=\left\{\begin{array}{c} 1,\;if\;u_{ij}\leq T\\ 0,\;if\;u_{ij}>T \end{array}\right.\\ {AC}_{ij}=\sum_{c}{(a}_{cj}\cdot h_{ij})\\ {AC}_{i}=\sum_{j}{AC}_{ij} \end{array}$$

(4) $$\begin{array}{l} {f_{ij}=e}^{-(k\cdot u_{ij})}\\ {AP}_{ij}=\sum_{c}\left(a_{cj}\cdot f_{ij}\right)\\ {AP}_{i}=\sum_{j}{AP}_{ij} \end{array}$$

where *u_ij_* is the mean travel time from *z_i_* to activity zone *z_j_*, *T* is the travel time threshold, *a_cj_* is the total number of activities of type *c* in *z_j_*, *h_ij_* is the binary function, and *f_ij_* is the impedance function with *k* being the controlling parameter.

##### The New Measure

Assume that across all observed trips for *z_ij_*, there are *n* discrete travel times (*t*_1_, …, *t_n_*) with corresponding probabilities (*p*_1_, …,*p_n_*), such that *p*_1_ + … + *p_n_* = 1. Based on Formula (4), the impedance function *f’_ij_*, describing the average effect across all the times, is given as

(5) $$\;{f^{\prime }}_{ij}={p_{1}e}^{-(k\cdot t_{1})}+{p_{2}e}^{-(k\cdot t_{2})}\ldots +{p_{n}e}^{-(k\cdot t_{n})}$$

The discrete distribution of travel times can be replaced with a continuous probability density function *P_ij_*(*t*). Using *f_t_ = e^−kt^*, the new impedance function *g_ij_* for *z_ij_*, measures *AN_ij_* for *z_ij_* and *AN_i_* for *z_i_* are defined as

(6) $$\begin{array}{l} g_{ij}=\frac{\int_{t_{\min}}^{t_{\max}}P_{ij}(t)\cdot f_{t}\cdot d(t)}{\int_{t_{\min}}^{t_{\max}}P_{ij}(t)\cdot d(t)}=\frac{\int_{t_{\min}}^{t_{\max}}P_{ij}(t)\cdot e^{-kt}\cdot d(t)}{\int_{t_{\min}}^{t_{\max}}P_{ij}(t)\cdot d(t)}\\ {AN}_{ij}=\sum_{c}\left(a_{cj}\cdot g_{ij}\right)\\ {AN}_{i}=\sum_{j}{AN}_{ij} \end{array}$$

Here, *t_min_* and *t_max_* represent the minimum and maximum travel times for *z_ij_*, respectively, and *f_t_* reflects the impact of travel times *t*. The new measure *AN_i_* integrates the existing measure *AP_i_* with the probability distribution *P_ij_*(*t*) of travel times for all trips between each pair of study and activity zones. The key difference lies in the impedance function; for *AP_i_*, *f_ij_* only accounts for the effect (and accessibility) of the mean travel time *u_ij_*, ignoring individual times. In contrast, *AN_i_* calculates the effect *f_t_* for each individual travel time and uses *g_ij_* to represent the average of *f_t_* across all the times.

**Figure 2:**
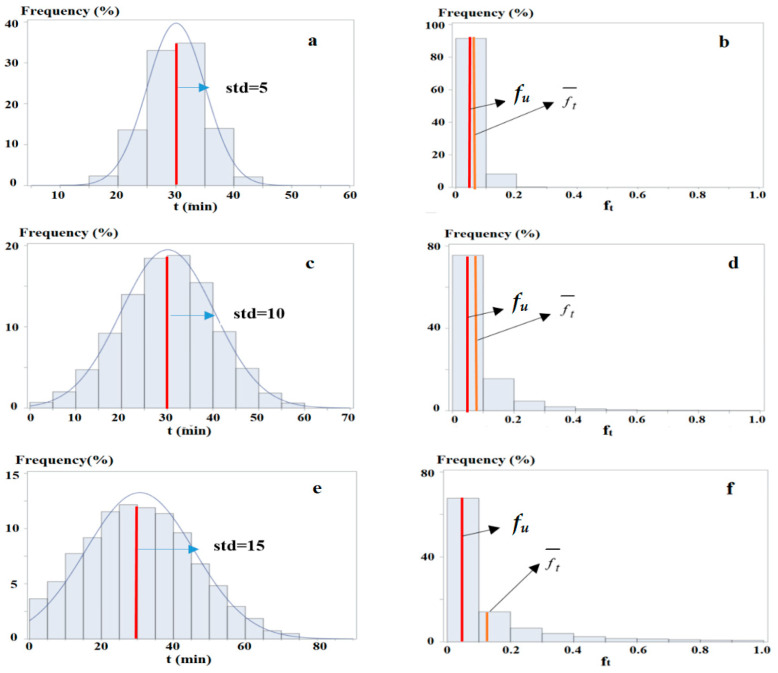
An example of varied accessibility for zone pairs with different travel time distributions. Note: In (**a**,**c**,**e**), the *x*-axis denotes travel times, *std* is the standard deviation, and the blue curves and red lines represent the fitted normal distributions and values of *u_ij_*. In (**b**,**d**,**f**), the *x*-axis denotes values of *f_t_* = *e^−kt^*, and the red and orange lines indicate *f_u_* = *f_ij_* and $\bar{f_{t}}$, respectively.

Figure 2 illustrates the difference between *g_ij_* and *f_ij_*. In this figure, *z*_1_ and *z_2_* represent two study zones, and *z_j_* is an activity zone. The travel time *t* from *z*_1_ to *z_j_* (i.e., *z*_1*j*_) (Figure 2a), and from *z_2_* to *z_j_* (i.e., *z_2j_*) (Figure 2c) follows normal distributions with identical mean values (*u_ij_* = 30 min) but different standard deviations (*std_ij_* = 5 and *std_ij_* = 10, respectively). Compared with *z*_1*j*_, *z_2j_* exhibits a more dispersed travel time distribution, resulting in more trips with shorter (e.g., *t* < 10 min) and longer (e.g., *t* > 50 min) times.

**Figure 3:**
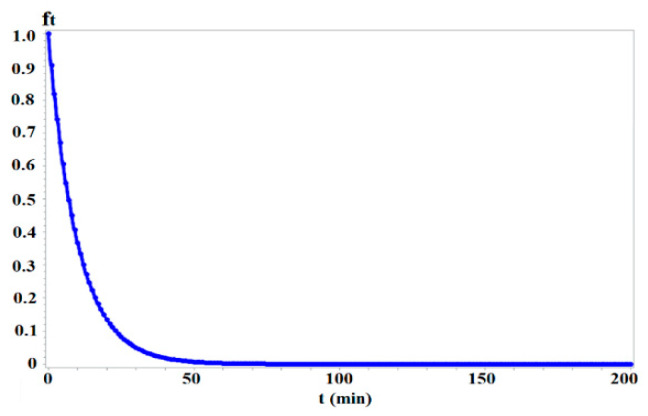
The negative exponential function *f_t_* = *e^−kt^*. Note: *k* = 0.1.

Given that *f_t_* = *e^−kt^* (Figure 3) yields higher values for shorter times, *z_2_* achieves a higher accessibility level than *z*_1_. This is evidenced by the average of *f_t_ = e^−kt^* (i.e., $\bar{f_{t}}$, equal to *g_ij_*) over all trips, calculated as 0.06 for *z*_1_ (Figure 2b) and 0.08 for *z_2_* (Figure 2d), with *k* = 0.1. This demonstrates that, under the *NEF* framework, accessibility is more influenced by trips with shorter travel times, as shorter times lead to less diminishing attractiveness and higher accessibility.

Figure 2e depicts the travel time distribution from a third zone *z*_3_ to *z_j_*, with the same mean (*u_ij_* = 30 min) but an even larger standard deviation (*std_ij_* = 15). This generates even more trips with shorter times, further increasing accessibility to 0.12 (Figure 2f).

For all the three zones, the existing measure *f_ij_* = 0.05, which is smaller than *g_ij_* for each zone. The differences are −0.01, −0.03, and −0.07, corresponding to underestimations of accessibility of these zone (pairs) by 17%, 38%, and 58%, respectively. This highlights that *f_ij_* underestimates accessibility more significantly as *std_ij_* increases, revealing that greater variability in travel times results in higher accessibility underestimated by the existing measure.

#### 2.2.4. Zones with the Lowest Level of Accessibility Detection

Using *AN_i_*, all study zones are sorted in ascending order, generating a rank *ANR_i_* for each zone. A percentage *TH_Per_* of zones with the lowest ranks is identified as the set *LowZone_AN_*, representing areas with the lowest accessibility. For comparison, the zones are also ranked by *AP_i_* and *AC_i_*, forming ranks *APR_i_* and *ACR_i_*, and corresponding sets *LowZone_AP_* and *LowZone_AC_* of low accessibility zones. Variations between these rankings and sets are analysed, highlighting the added value of *AN_i_*.

## 3. Results

### 3.1. Passenger Trips

**Figure 4:**
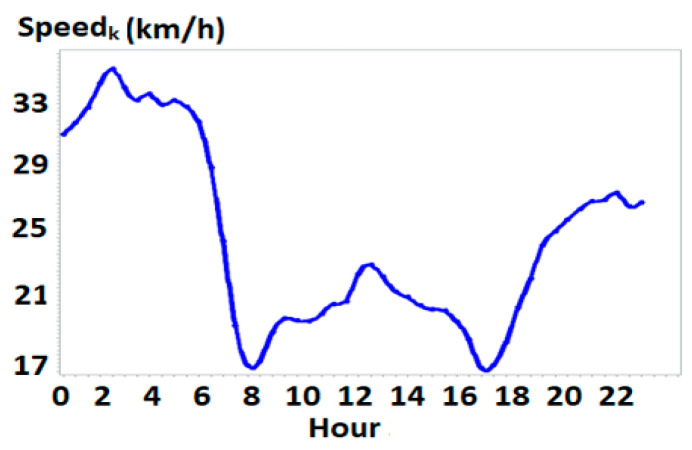
Distribution of average speeds in each half an hour of a weekday. Note: the *x*-axis denotes the time of the day, and the *y*-axis is the average speed of trips over half-hour intervals.

The speed threshold was set at *TH_Speed_* = 120 km/h (the maximum speed limit in China) to extract passenger trips, yielding 478,026 trips per day. Figure 4 illustrates the distribution of average speeds (*Speed_k_*) for trips over half-hour intervals across weekdays, showing clear variations in driving speeds throughout the day. Based on this distribution, we divided a day into four periods: morning (7–9 AM), daytime (9 AM–16 PM), evening (16–18 PM) (evening), and night (18 PM–7 AM), with corresponding average speeds of 18.4, 21.4, 18.8, and 27.3 km/h, respectively.

### 3.2. Travel Pattern Matrices and Study Zones

The city was divided into *GridX* × *GridY* zones for the travel pattern matrix, where larger grid dimensions improve spatial resolution but reduce the number of observed trips between zones. In order to achieve statistically sound results, we specified *GridX* = *GridY* = 40, resulting in a total of 1600 zones, each being 1.87 km^2^ in size. For comparison, grid sizes in other studies range from 0.15 km^2^ in Denizli, Turkey, for public transit studies [10] to 2.14 km^2^ in Twin Cities, MN, USA, for car-based accessibility analysis [9].

The travel pattern matrix *OD* (*z_i_*, *z_j_*, *TimeP*, *Day*, *DayT*) was constructed using this spatial partitioning and the four temporal periods, with *i*, *j* = 1600, *TimeP* = 4, *Day* = 66 (Weekdays) and 26 (Weekends) and *DayT* = Weekdays and Weekends. This study focused on weekday mornings, but the methodology can be extended to other periods and weekends.

Study zones and passenger travel times were extracted using thresholds *TH_M_* = 20, *TH_r_* = 3.32 (i.e., the 95th percentile of circuity of all trips over the zone pairs) and *TH_t_* = *u_ij_* + *3std_ij_* (*u_ij_* and *std_ij_* being the mean and standard deviation of each pair *z_ij_*). Trips with *t* > *u_ij_* + *3std_ij_*, which only occur at the probability of 0.003, were excluded as abnormal trips. This process identified 103 study zones and 152 activity zones, representing 6.4% and 9.5% of the total grids. Each study zone recorded at least 20 trips departing in the morning and arriving at night, while each activity zone contained at least one activity location. The minimum number of trips between the study and activity zones was 78.

### 3.3. Accessibility Computation

Two parameters were used to compute accessibility measures: the probability density function *P_ij_*(*t*) of travel times and the controlling parameter *k* in the impedance function *NEF*. Statistical tests were conducted using the Kolmogorov–Smirnov method [33], which confirmed that *P_ij_*(*t*) follows a normal distribution, with *p*-values for all the concerned zone pairs ranging from 0.8 to 0.99 (well above the 0.05 threshold). In addition, different values of *k* have been employed in the literature [15,19,21,34], subject to the study area and type of activities. In this experiment, we adopted the commonly used value of *k* = 0.1 for the integrated analysis of all the activity types [19,21]. Activity attractiveness in each activity zone is quantified based on the total number of activities of all types in the zone.

### 3.4. Comparison Between AN_i_ and AP_i_

#### 3.4.1. Impedance Functions *g_ij_* and *f_ij_*

The correlation coefficient between the new impedance function *g_ij_* and the traditional impedance function *f_ij_* is 0.97, indicating a strong overall positive relationship. Nevertheless, significant variations exist between individual pairs. On average, *f_ij_* underestimates accessibility by an absolute value of 0.02 and a relative percentage of 28% (Δ*f_ij_* = *f_ij_* − *g_ij_*; *әf_ij_* = Δ*f_ij_*/*g_ij_*).

**Figure 5:**
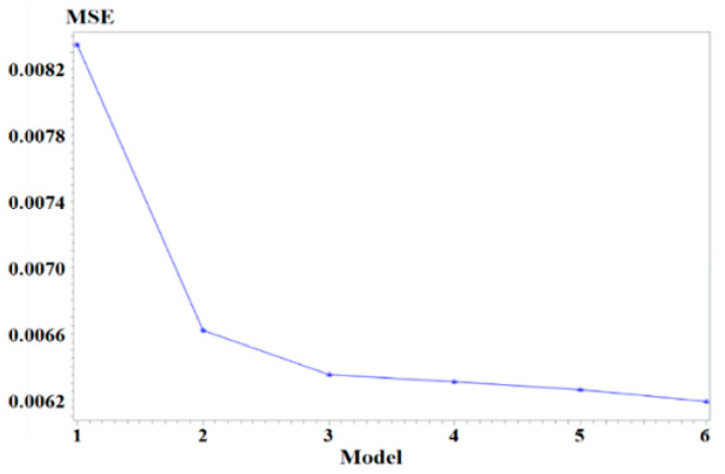
MSE of the models at major steps. Note: The *x*-axis represents the models (Model_1_–Model_6_) at each step (1–6), and the *y*-axis denotes *MSE*. The variables contained in Model_1_–Model_6_ are *r_ij_*, (*r_ij_*, *u_ij_*), (*r_ij_*, *u_ij_*, *skew_ij_*), (*r_ij_*, *u_ij_*, *skew_ij_*, *skew_ij_/u_ij_*), (*r_ij_*, *u_ij_*, *skew_ij_*, *skew_ij_/u_ij_*, *std_ij_·skew_ij_*), (*r_ij_*, *u_ij_*, *skew_ij_*, *skew_ij_/u_ij_*, *std_ij_·skew_ij_*, *skew_ij_·u_ij_*_,_ *std_ij_*, *u_ij_·std_ij_*, *std_ij_/skew_ij_*), respectively, where *r_ij_* = *std_ij_/u_ij_*.

To investigate the factors contributing to the varying degrees of underestimations, we analysed the relationship between Δ*f_ij_* and the travel time distributions of the corresponding zone pairs using stepwise regression modelling techniques [35]. In this process, each of the variables including *u_ij_*, *std_ij_* and skewness *skew_ij_*, or each combination of these variables was added to the model at each step, and the variable (or variable combination) that led to the least mean squared error (*MSE*) was chosen. Figure 5 presents *MSE* of the obtained models at each major step, showing that *MSE* decreases sharply from Model_1_–Model_3_ but declines slowly over Model_4_–Model_6_. Model_3_ was thus selected as the final model. Its equation is given in Formula (7), and it achieves an *MSE* of 0.006. *MSE* and *skew_ij_* are computed according to Formula (8).

According to Model_3_, the mean (*u_ij_*), the skewness of the distribution (*skew_ij_*) and the ratio of the standard deviation to the mean (*r_ij_* = *std_ij_*/*u_ij_*) are key factors. Specifically, shorter times *u_ij_*, higher skewness *skew_ij_*, and larger ratios *r_ij_* are associated with greater differences between *f_ij_* and *g_ij_*, leading to a more significant underestimation of accessibility by *f_ij_*.

(7) $${\Delta f}_{ij}\;\sim -0.001+0.0003\cdot u_{ij}-0.0009\cdot {skew}_{ij}-0.0889\cdot r_{ij}$$

(8) $$\begin{array}{l} {skew}_{ij}=\frac{\frac{1}{m_{ij}}\cdot \sum_{t}{\left(t-u_{ij}\right)}^{3}}{{\left[\frac{1}{m_{ij}}\cdot \sum_{t}{\left(t-u_{ij}\right)}^{2}\right]}^{3/2}}\\ MSE=\frac{\sum_{i,j}{{(\Delta f}_{ij}-\Delta {\hat{f}}_{ij})}^{2}}{N_{pair}} \end{array}$$

Here, *m_ij_* denotes the number of trips for *z_ij_*, while *N_pair_* and $\Delta {\hat{f}}_{ij}$ are the numbers of all the pairs and the predicted value of Δ*f_ij_*, respectively.

To further characterise the distinctions between *g_ij_* and *f_ij_*, we introduced an additional variable *prop_ij_* for each pair *z_ij_*, as defined in Formula (9).

(9) $$\begin{array}{l} {prop}_{ij}=\frac{N(t\geq t_{eq})}{N(t_{\min}\leq t\leq t_{\max})},\;with\\ \frac{\int_{t_{eq}}^{t_{\max}}P_{ij}(t)\cdot f_{t}\cdot d(t)}{\int_{t_{eq}}^{t_{\max}}P_{ij}(t)\cdot d(t)}=\frac{\int_{t_{eq}}^{t_{\max}}P_{ij}(t)\cdot e^{-kt}\cdot d(t)}{\int_{t_{eq}}^{t_{\max}}P_{ij}(t)\cdot d(t)}=f_{ij} \end{array}$$

Here, *t_min_* and *t_max_* are the shortest and longest travel times for *z_ij_*, respectively, *t_eq_* (*t_min_* < *t_eq_* < *t_max_*) is the time point at which the mean of *NEF* (*f_t_*) over *t* ϵ [*t_eq_*, *t_max_*] equals *f_ij_*, and *N*(*t* ≥ *t_eq_*) and *N*(*t_min_* ≤ *t* ≤ *t_max_*) are the numbers of trips with *t* ≥ *t_eq_* and *t_min_* ≤ *t* ≤ *t_max_*. *Prop_ij_* represents the proportion of trips with the longest travel times for which the mean value of *f_t_* equals *f_ij_*. This implies that *f_ij_* effectively accounts for the accessibility of only a subset of trips for each zone pair. Further statistics reveal that *prop_ij_* varies between 0.61–0.98, averaging 0.76, indicating that traditional measures accommodate only 76% of trips (on average) with the longest travel times.

**Figure 6:**
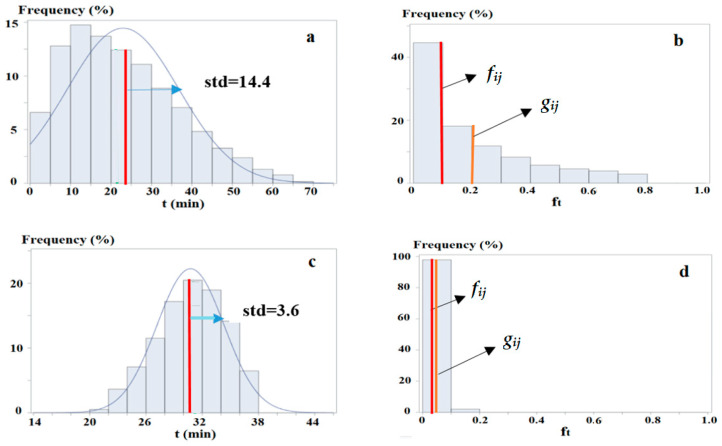
Travel time distributions and values of *f_ij_* and *g_ij_* of two actual zone pairs. Note: In (**a**,**c**), the *x*-axis denotes travel times, *std* is the standard deviation, and the blue curves and red lines represent the fitted normal distributions and values of *u_ij_*. In (**b**,**d**), the *x*-axis denotes values of *f_t_ = e^−kt^*, and the red and orange lines indicate *f_ij_* and *g_ij_*, respectively.

Figure 6 presents two actual zone pairs, *z*(21,20)⟶*z*(20,19) and *z*(23,15)⟶*z*(23,24), which exhibit the largest (Δ*f_ij_* = −0.1) and a relatively smaller (Δ*f_ij_* = −0.003) difference, respectively. The first pair (Figure 6a) has a relatively short mean travel time (23.3 min) but a large deviation (14.4), generating large values of *r_ij_* (0.62) and skewness (0.82). This leads to *f_ij_* underestimating the accessibility by 51%, covering only 74% of trips (Figure 6b). In contrast, the second pair (Figure 6c) have comparatively long travel times (an average of 30.8 min) but smaller deviations (3.6), yielding lower values of *r_ij_* (0.12) and skewness (−0.32). Consequently, *f_ij_* underestimates the accessibility only by 6% while encompassing 94% of trips (Figure 6d).

#### 3.4.2. Accessibility Measures *AN_i_* and *AP_i_*

Let Δ*AP_i_* = *AP_i_ − AN_i_* and *әAP_i_* = Δ*AP_i_*/*AN_i_*. The values of Δ*AP_i_* and *әAP_i_* range between −42 and −505 and between −0.06 and −0.28, respectively, with averages of −273 and −0.17. This indicates that, due to the underestimation by *f_ij_*, *AP_i_* underestimates the accessibility of each zone *z_i_* by an absolute average value of 273 and a relative proportion of 17% when compared to *AN_i_*. Moreover, for each *z_i_*, the average of *prop_ij_* over all the activity zones ranges from 82% to 89%, showing that *AP_i_* accounts for only 82–89% of trips (with the longest travel times) between the study zone and each activity zone.

#### 3.4.3. Accessibility Ranks *ANR_i_* and *APR_i_*

Despite *AP_i_* (and *f_ij_*) underestimating accessibility across all study zones (and zone pairs), the degrees of underestimations vary, leading to differences between the existing rank *APR_i_* and the new rank *ANR_i_*. Specifically, let Δ*APR_i_* = *APR_i_* − *ANR_i_*. Among all zones, 40% have an *APR_i_* rank lower than the corresponding *ANR_i_* rank, 42% have a higher *APR_i_* rank, and the remaining 18% have equal ranks (Δ*APR_i_* = *0*). The minimum and maximum values of Δ*APR_i_* are −10 and 12, respectively.

To identify zones suffering from the lowest accessibility, a threshold *TH_Per_* was defined, tailored to the urban area’s specific conditions (e.g., general accessibility levels and the severity of accessibility issues under investigation) [30]. In this study, *TH_Per_* = 20% was set, resulting in 20 zones with the lowest ranks (i.e., *ANR_i_* ≤ 20 or *APR_i_* ≤ 20). These zones form the sets *LowZone_AN_* and *LowZone_AP_*, referred to as the *problematic zones*. The mean accessibility measures *AN_i_* and *AP_i_* per study zone are 1534 and 1423, respectively, while the largest values of *AN_i_* and *AP_i_* in *LowZone_AN_* and *LowZone_AP_* are 643 and 590, constituting only 41.9% and 41.5% of the corresponding mean measures.

**Figure 7:**
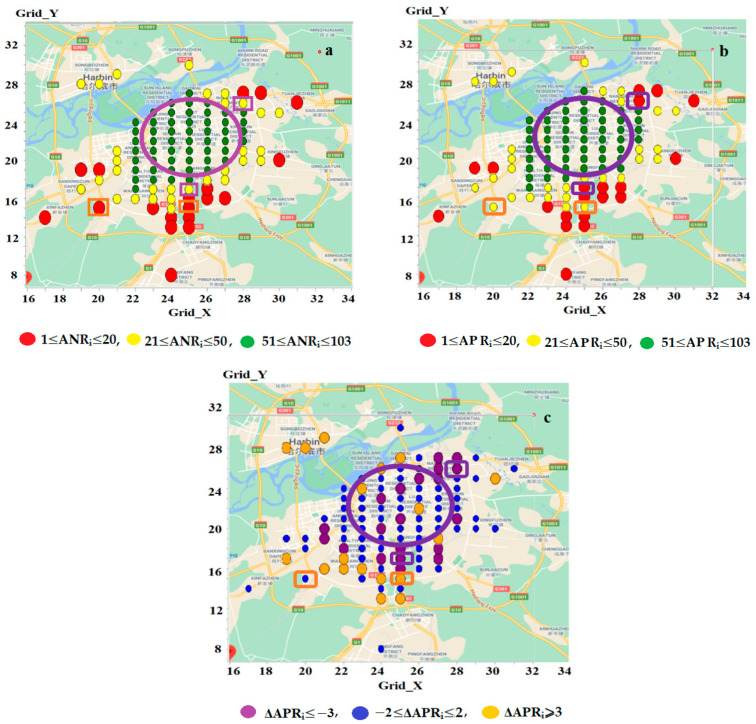
Geographic distributions of study zones with *ANR_i_* (**a**), *APR_i_* (**b**) and Δ*APR_i_* (**c**), respectively. Note: In (**a**,**b**), the large filled red, small filled yellow and green circles represent zones with *ANR_i_* and *APR_i_* ranks of 1–20, 21–50 and 51–103, respectively. In (**c**), the large filled purple, small filled blue and large filled orange circles represent zones with Δ*APR_i_* ≤ −3, −2 ≤ Δ*APR_i_* ≤ 2, and Δ*APR_i_* ≥ 3, respectively. In all the figures, the zones enclosed in purple rectangles are included in *LowZone_AP_* but not in *LowZone_AN_*; while those enclosed in orange rectangles are in *LowZone_AN_* but not in *LowZone_AP_*. The large unfilled purple oval outlines *Area_cen_*.

Figure 7a,b illustrate the geographic distributions of all study zones, where large filled red, small filled yellow and green circles represent zones with *ANR_i_* and *APR_i_* ranks of 1–20, 21–50 and 51–103, respectively. Comparing problematic zones identified by the two measures reveals that two zones (*z*(25,17) and *z*(28,26), enclosed in purple rectangles) are included in *LowZone_AP_* (with *APR_i_* = 18 and 17) but not in *LowZone_AN_* (*ANR_i_* = 28 and 26). Conversely, two other zones, (*z*(20,15) and *z*(25,15), enclosed in orange rectangles), are found in *LowZone_AN_* (*ANR_i_* = 20 and 18) but not in *LowZone_AP_* (*APR_i_* = 22 and 23). Thus, of the 20 problematic zones, 16 (80%) are identified by both measures, while the remaining 4 (20%) are assessed differently.

#### 3.4.4. Geographic Features

To examine the geographic features influencing ranking differences, we classified all study zones into three categories based on Δ*APR_i_*: Δ*APR_i_* ≤ −3, −2 ≤ Δ*APR_i_* ≤ 2, and Δ*APR_i_* ≥ 3. These categories are represented in Figure 7c, which shows that most zones with a lower *APR_i_* rank (Δ*APR_i_* ≤ −3, represented by large filled purple circles) are located in or around the urban centre (*Area_cen_*) where a high concentration of activities is established. In contrast, zones with a higher *APR_i_* rank (Δ*APR_i_* ≥ 3, represented by large filled orange circles) are predominantly found in suburban areas away from *Area_cen_*. This geographic pattern can be attributed to the differences in traffic conditions and travel time distributions. For zones *z_i_* near *Area_cen_*, travel times between *z_i_* and activity zones *z_j_*—particularly those within *Area_cen_*—are generally short (e.g., due to proximity) but exhibit significant variability (e.g., due to congestion). This results in small mean travel times (*u_ij_*) but large values of deviations (*std_ij_*), ratios (*r_ij_*), and skewness (*skew_ij_*) [21]. Consequently, *f_ij_* < *g_ij_* and *AP_i_* < *AN_i_* to a greater extent, leading to *z_i_* being assigned a lower *APR_i_* rank compared to its corresponding *ANR_i_* rank. Conversely, zones located far from *Area_cen_* typically experience longer travel times between *z_i_* and *z_j_* (especially to activity zones within *Area_cen_*) but with less variability (e.g., due to reduced congestion in suburban areas). These conditions produce travel time distributions with larger *u_ij_* but relatively smaller *std_ij_*, *r_ij_*, and *skew_ij_*. As a result, *f_ij_* < *g_ij_* and *AP_i_* < *AN_i_* to a lesser extent, causing *z_i_* to receive a higher *APR_i_* rank relative to its *ANR_i_* rank).

### 3.5. Comparison Between AN_i_ and AC_i_

In addition to *AP_i_*, another widely used location-based measures is the contour measure *AC_i_*. In this subsection, we conducted a final comparison between *AN_i_* and *AC_i_*. To this end, *AC_i_* was computed for each study zone (according to Formula (3)) with the typical threshold value of *T* = 30 min [9]. The corresponding ranks *ACR_i_* were then derived, and a set *LowZone_AC_*, containing 20 zones with the lowest *ACR_i_* ranks, was identified.

#### 3.5.1. *AN_i_* and *AC_i_*

In relation to *g_ij_*, the binary function *h_ij_* (used in *AC_i_*) either overestimates or underestimates the accessibility of a zone pair, whereas *f_ij_* consistently underestimates all pairs. Specifically, for 49% of zone pairs where *u_ij_ ≤* 30 min, *h_ij_* = 1, leading to an overestimation of accessibility by 22–2610%, with an average of 670% (0.22 ≤ *әh_ij_* ≤ 26.1, *әh_ij_* = (*h_ij_-g_ij_*)*/g_ij_*). Conversely, for the remaining 51% of pairs where *u_ij_* > 30 min, *h_ij_* = 0, resulting in an underestimation of accessibility by 100% (*әh_ij_* = −1). The overall correlation between *g_ij_* and *h_ij_* across all pairs is 0.66, displaying a much lower correlation than between *g_ij_* and *f_ij_* (0.97).

Despite the over- or underestimation, *AC_i_* overestimates the accessibility of each zone by 68–509%, with an average of 360% (0.68 ≤ *әAC_i_* ≤ 5.09, *әAC_i_* = (*AC_i_* − *AN_i_*)/*AN_i_*). This demonstrates a distinct feature of *AC_i_*, contrasting with *AP_i_*, which underestimates accessibility for all zones.

#### 3.5.2. *ANR_i_* and *ACR_i_*

Significant variations also exist in the accessibility rankings. Specifically, 50% of zones have a lower *ACR_i_* rank than the corresponding *ANR_i_*, 44% have a higher *ACR_i_* rank, and only 7% have equal ranks. The differences between these two rankings (Δ*ACR_i_* = *ACR_i_* − *ANR_i_*) range from −18 to 18, which is greater than the range observed for Δ*APR_i_* (−10 to 12).

**Figure 8:**
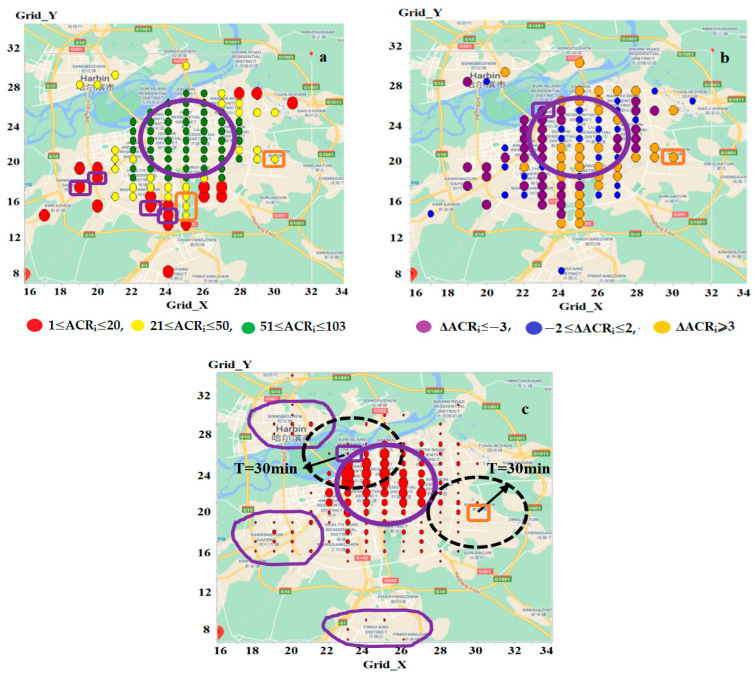
Geographic distributions of study zones with *ACR_i_* (**a**), Δ*ACR_i_* (**b**) and activity zones (**c**). Note: In (**a**), the large filled red, small filled yellow and green circles represent zones with *ACR_i_* ranks of 1–20, 21–50 and 51–103, respectively. The zones enclosed in purple rectangles are included in *LowZone_AC_* but not in *LowZone_AN_*; while those enclosed in orange rectangles are in *LowZone_AN_* but not in *LowZone_AC_*. In (**b**), the large filled purple, small filled blue and large filled orange circles represent zones with Δ*ACR_i_* ≤ −3, −2 ≤ Δ*ACR_i_* ≤ 2, and Δ*ACR_i_* ≥ 3, respectively. In (**c**), the filled red circles denote activity zones, with the radius being proportional to the number of activities. Red circles enclosed by purple polygons outline activities in the northwest, southwest and south. The two black dash circles represent *Area*(*z_i_*,*T*) for *z*(23,25) and *z*(30,20). In (**b**,**c**), the zones enclosed by purple and orange rectangles are the exemplified zones *z*(23,25) and *z*(30,20), featuring a lower and higher *ACR_i_* rank, respectively. In all the figures, the large unfilled purple oval outlines *Area_cen_*.

When comparing problematic zones in *LowZone_AC_* (Figure 8a) with those in *LowZone_AN_* (Figure 7a), four zones (*z*(19,17), *z*(20,18), *z*(23,16), and *z*(24,15), highlighted by large filled red circles in purple rectangles) are included in *LowZone_AC_* but not in *LowZone_AN_* (*ACR_i_* = 12, 19, 20 and 18, while *ANR_i_* = 22, 33, 29 and 21, respectively). Conversely, four other zones (*z*(25,14), *z*(25,15), *z*(25,16), and *z*(30,20), highlighted by small filled yellow circles in orange rectangles) are present in *LowZone_AN_* but excluded from *LowZone_AC_* (*ANR_i_* = 8, 18, 15 and 12, while *ACR_i_* = 26, 36, 25 and 27, respectively). Thus, only 12 (60%) are identified as problematic by both measures, while the remaining 8 (40%) are evaluated differently. The level of detection consistency (i.e., 60%) is lower than that identified by *ANR_i_* and *APR_i_* (i.e., 80%).

#### 3.5.3. Geographic Features

Figure 8b visualises the geographic distribution of zones classified into three categories based on Δ*ACR_i_*: Δ*ACR_i_* ≤ −3, −2 ≤ Δ*ACR_i_* ≤ 2, and Δ*ACR_i_* ≥ 3. Zones with lower *ACR_i_* ranks (represented by purple circles) are predominantly located in the western part of the city. In contrast, those featuring a higher *ACR_i_* rank (represented by orange circles) are mostly found in the eastern part. Further investigations reveal that this geographic tendency is closely linked to the distributions of activities in the urban area, as shown in Figure 8c, where filled red circles represent activity zones with the radius being proportional to the number of activities. Of these activity zones, 94.6% are established in *Area_cen_* and its surrounding area, while the remaining 1.6%, 2.9% and 0.9% are built in the northwest, southwest and south of the city (i.e., in the purple polygons), respectively. Notably, no activities are found in the eastern part. This uneven distribution explains the observed ranking differences. A zone in the west (e.g., *z*(23,25), enclosed by the purple rectangle) has more activities within the neighbourhood of the area Area(*z_i_*,*T*) (i.e., *places* reached from *z_i_* within the travel time *T*). Conversely, a zone in the east (e.g., *z*(30,20), enclosed by the orange rectangle) has fewer activities within or outside *Area*(*z_i_*,*T*), leading to a higher *ACR_i_* rank. The dashed black circles in Figure 8c represent *Area*(*z_i_*,*T*) for *z*(23,25) and *z*(30,20), respectively.

This geographic pattern is further influenced by the modelling methods. *AC_i_* calculates accessibility using a discrete and binary manner; each activity zone *z_j_* is classified as either *u_ij_* ≤ T or *u_ij_* > *T*. Zones with *u_ij_* ≤ *T* form *Area*(*z_i_*,*T*), and *AC_i_* estimates accessibility based solely on the total number (or size) of activities within *Area*(*z_i_*,*T*), disregarding activities outside this area. This makes *AC_i_* only dependent on the value of *T* and activity situations inside *Area*(*z_i_*,*T*). In comparison, *AN_i_* (or *AP_i_*) treats travel times as a continuous variable and models the effects of the times in a continuous way. Particularly, *AN_i_* (or *AP_i_*) computes accessibility by considering all activities in the urban area and using *k* to control the weights of travel times on activity attractiveness. Thus, *AN_i_* (or *AP_i_*) is not only related to the activity conditions in *Area*(*z_i_*,*T*), but also influenced by the activity distributions outside this region. This underlines the above ranking differences between *ANR_i_* and *ACR_i_*. For a zone (e.g., *z*(23,25)), if more activities are located outside *Area*(*z_i_*,*T*), *AN_i_* tends to be larger and *ANR_i_* be higher, resulting in *ACR_i_* being lower than the corresponding *ANR_i_*. Conversely, for a zone like *z*(30,20), which has fewer activities outside *Area*(*z_i_*,*T*), *AN_i_* is smaller, leading to a lower *ANR_i_* rank and a higher *ACR_i_* rank.

## 4. Discussion

Achieving equitable transport accessibility and a balanced distribution of urban services is one of the primary objectives of transport managers and urban planners [5]. As cities grow and populations expand, accessibility challenges become increasingly critical, necessitating more advanced and accurate analysis methods [1]. To address this challenge, we have developed a novel approach to measure accessibility to various urban services by car. Compared to traditional methods, this new approach is more sensitive to traffic conditions and travel time distributions, providing a more objective representation of accessibility. Additionally, the continuous generation of GPS data from urban vehicles enables timely updates of the derived results, allowing these results to keep pace with rapid urban land-use changes, population growth, and evolving mobility patterns.

### 4.1. Major Differences Between the New and Existing Measures

When the proposed method was applied to the study city, a certain level of deviation was observed between the new measure, *AN_i_*, and existing measures, *AP_i_* and *AC_i_*. Specifically, compared to *AN_i_*, *AP_i_* underestimates accessibility across all the study zones by an average of 17%, whereas *AC_i_* overestimates accessibility by 360%. Moreover, under- or overestimation varies across zones, leading to discrepancies between the new ranking, *ANR_i_*, and existing rankings, *APR_i_* and *ACR_i_*. The geographic characteristics and underlying causes for these discrepancies also differ. The differences between *ANR_i_* and *APR_i_* are primarily influenced by traffic conditions and travel time variations. Most zones with a lower *APR_i_* rank are located in or around the urban centre, *Area_cen_*, where a high concertation of activities is established (See Figure 7c). In these zones (e.g., *z_i_*), travel times between *z_i_* and activity zones *z_j_*—particularly those within *Area_cen_*—are generally short due to proximity but exhibit high variability due to congestion. As a result, travel time distributions show large deviations (*std_ij_*), skewness (*skew_ij_*), and/or ratios (*r_ij_* = *std_ij_*/*u_ij_*). The larger travel time deviations in *z_i_* result in more trips with shorter times (relative to the mean), leading to a lower reduction in attractiveness and, therefore, a higher level of accessibility represented by *AN_i_* and a higher rank by *ANR_i_* (compared to *AP_i_* and *APR_i_*). In contrast, zones with a higher *APR_i_* rank are predominantly found in suburban areas farther from *Area_cen_*. These zones (e.g., *z_i_*) typically experience longer travel times between *z_i_* and *z_j_* (especially within *Area_cen_*) but with less variability (e.g., due to reduced congestion in suburban areas). Under such conditions, travel time distributions have larger *u_ij_* values but relatively smaller *std_ij_*, *skew_ij_* and *r_ij_*, meaning that travel times are more concentrated around the mean travel time. This results in fewer trips with shorter travel times, leading to a greater reduction in activity attractiveness and, consequently, a lower level of accessibility as reflected by *AN_i_* and a lower rank by *ANR_i_* (compared to *AP_i_* and *APR_i_*).

The differences between *ANR_i_* and *ACR_i_* are more closely related to the spatial distribution of activities within and outside the *Area*(*z_i_*,*T*) (places reached from *z_i_* within *T*) (See Figure 8c). In zones with a larger number of activities located outside *Area*(*z_i_*,*T*), *AN_i_* tends to be larger, resulting in a higher *ANR_i_* and a lower *ACR_i_*. Conversely, in zones with fewer activities outside *Area*(*z_i_*,*T*), *AN_i_* is relatively smaller, leading to a lower *ANR_i_* and a correspondingly higher *ACR_i_*.

The above discrepancies highlight the inaccuracies of the existing measures *AP_i_* and *AC_i_* and underscore the advantages of adopting the new measure *AN_i_*, particularly in urban environments characterised by heavy congestion surrounding *Area_cen_* and uneven activity distributions outside *Area_cen_*. The higher the congestion levels and the more uneven the activity distributions, the larger the disparities between the existing and new measures. In the experimental city, the minimum and maximum differences between *APR_i_* and *ANR_i_* range from −10 to 12, while those between *ACR_i_* and *ANR_i_* span from −18 to 18. Across all the 103 study zones, this ranking variation translates into relative percentage changes: a shift of −10% to +12% between *APR_i_* and *ANR_i_*, and −17% to +17% between *ACR_i_* and *ANR_i_*.

### 4.2. Potential Applications of the New Method

The proposed method facilitates systematic analysis of accessibility across urban road networks and the identification of zones experiencing significant (vehicle-based) accessibility problems. It can also be applied to assess the impact of implemented land-use or transport policies on accessibility by comparing GPS-derived measures before and after their adoption. Across these applications, the new measure *AN_i_* provides a more accurate reflection of actual network performance by considering average travel times and detailed travel time distributions. This allows for more precise accessibility evaluations and problem identification, aiding in the design of policies better aligned with real-world traffic conditions, thereby enhancing network reachability and reducing inequities.

This is particularly relevant in the post-COVID era. During and after the pandemic, the rise in remote work has reduced commuting trips while increasing home-centred activities and travel [36,37]. Mobility patterns have shifted from work-centric destinations to local areas near homes, making the re-evaluation of road network accessibility, particularly in local areas, an essential step [38]. In this context, the proposed *AN_i_* measure is especially valuable for identifying areas with poor accessibility and assisting governments in addressing these shifts, ultimately improving reachability for local communities.

### 4.3. Future Research Avenues

Several avenues for future research remain open. The first concerns parameter sensitivity analysis. This method relies on a set of parameters, and the experimental results are influenced by their specified values. Future research should systematically explore how variations in these parameter values would affect the results in order to provide more precise guidance on optimal parameter selection. Particularly, for this analysis, we simply adopted a fixed threshold (*TH_Per_* = 20%) to identify zones with the lowest accessibility and compared these zones detected by the new and existing measures. For subsequent research, applying more refined data analysis techniques—such as clustering [30]—would allow for a multi-level classification of zones by accessibility, enabling more precise identification of the lowest-ranking zones. We also recommend externally validating these lowest-ranking zones against observed accessibility conditions and examining the policy implications of any identified problems.

Additionally, the parameter *k* captures the effect of travel times between zones on people’s behaviour [11]; the higher the value, the greater the decreasing effect of travel times. Future work could explore alternative values of *k* beyond the current setting. While varying *k* changes the absolute accessibility measures of each zone pair and zone, it may also alter zone rankings. Increasing *k* generally reduces overall attractiveness across all zones, but the reduction in *AN_i_* is smaller for zones with larger deviations. Those zones tend to generate more short trips, and a higher *k* amplifies the influence of short trips, which mitigates their attractiveness loss. As a result, as *k* increases, *AN_i_* decreases for all zones, but zones with larger deviations experience a relatively smaller decrease and therefore rise in zone rankings (*ANR_i_*).

Second, future research should examine alternative probability distributions. In the case study, travel times for each zone pair were assumed to follow a normal distribution. However, in a more congested urban network, travel time distributions can be more right-skewed; functions such as lognormal or gamma distributions may better represent these conditions. These skewed distributions tend to increase the average travel time (*u_ij_*), which would likely amplify the underestimation of accessibility by the traditional function (*f_ij_*) relative to the new function (*g_ij_*).

Third, activity attractiveness in each activity zone was quantified based solely on the total number of activities of all types, without considering other attributes such as sizes or specific activity types. Since the primary differences between the new and existing measures stem from the impedance functions (*g_ij_* and *f_ij_*), this study focused on examining the differences resulted from these functions. The method to quantify activity attractiveness is unlikely to significantly impact the compared results, as all the accessibility measures use the same attractiveness values. Nevertheless, this quantification method could be refined by including additional attributes (e.g., sizes or types) or by weighting attributes to reflect activities’ relative importance [15].

Fourth, when deriving Formula (7) to characterise the relationship between Δ*f_ij_* and travel time distributions, we used three key variables: *u_ij_*, *std_ij_* and *skew_ij_*. However, other factors—such as the difference between the mean and median—likely also affect this relationship. Future work could explore these additional variables to create a more precise regression model.

Fifth, our analysis concentrated in the weekday morning rush hour (7:00–9:00 AM), and compared *AN_i_* against two established dynamic measures—*AP_i_* and *AC_i_*—all derived from this same period. Future research could shrink the temporal period to a shorter (e.g., one-hour) interval, instead of the full two-hour window used here. Given that traffic conditions remain relatively consistent throughout the rush hour and travel time variability exists within each shorter interval, we expect any differences between the new and existing measures to mirror the trends observed in our current findings. Beyond adjusting the time window, the proposed method offers broader applicability to other periods like afternoons, evenings, or weekends. However—given the large differences in travel patterns and traffic conditions—applying it to these periods would yield distinct accessibility values and zonal rankings compared to our current results.

Lastly, future research should explore the use of expanded data sources. This study demonstrates the utility and benefits of the new approach using GPS data from taxis. However, this data source has inherent limitations; for example, high-density residential areas with accessibility issues may be overlooked if they receive limited taxi trips. This limitation could be mitigated by incorporating GPS data from other urban vehicles, such as ride-hailing services, private cars, buses and trucks [39], and from smartphones carried by individuals while walking or using public transport. Given the heavy reliance on public transit in major cities, applying the proposed method to bus GPS data is particularly crucial for accurately assessing and improving accessibility gaps. Additionally, for this proof of concept, we used the 2016 taxi dataset. Urban land-use and population have changed considerably since then—especially after the COVID-19 pandemic—so future work should apply this method to more recent data and compare the results. Combining these varied data sources will strengthen the model’s robustness, enabling more precise identification of contemporary accessibility challenges—not just across road networks, but also within public transit and pedestrian pathways [40]. Alongside expanding the data, we also plan to improve visualisation in future work. Using a more geographically oriented tool—such as ColorBrewer 2 (https://colorbrewer2.org/) (accessed on 4 May 2025)—would better display zones and their accessibility variations.

## Data Availability

The corresponding author will provide the information supporting the research study’s conclusions upon reasonable request.

## Appendix A

**Table A1:** Classification of activity types.

ID	Activity Type	ID	Activity Type	ID	Activity Type	ID	Activity Type
1	Hotel	5	financial centre	9	tourism site	13	news media
2	Restaurant	6	transportation hub	10	shop	14	leisure
3	Government	7	school and university	11	social service	15	hospital
4	Police station	8	filling station	12	communication	16	factory and company

**Table A2:** Major variables used in the computation process.

General Variables	
*z_i_*, *z_j_* and *z_ij_*	The study and activity zones and the zone pair from *z_i_* to *z_j_*.
*a_cj_* and *A_j_*	The total number of activities of type *c* and activities of all types in *z_j_*.
*T*	The travel time threshold.
*f_t_*	The negative exponential function (*NEF*) *f_t_ = e^−kt^*.
Variables for each zone pair	
*u_ij_*, *std_ij_* and *skew_ij_*	The mean, standard deviation and skewness of travel time distributions for *z_ij_*.
$\bar{f_{t}}$	The average effect over the effect of each individual travel time *t* for *z_ij_*.
*P_ij_*(t)	The probability density function of *t* for *z_ij_*.
*AC_ij_*, *AP_ij_* and *AN_ij_*	The existing contour, potential and new measures for *z_ij_*, respectively.
*h_ij_*, *f_ij_* and *g_ij_*	The existing binary, impedance and new functions for *z_ij_*, respectively.
Δ*f_ij_*, *әf_ij_*, Δ*h_ij_* and *әh_ij_*	The absolute and relative differences between *f_ij_* and *g_ij_* as well as between *h_ij_* and *g_ij_*.
*prop_ij_*	The ratio between the number of trips over which the mean of *f_t_* is equal to *f_ij_* and the number of all trips from *z_ij_*.
Variables for each zone	
*AC_i_*, *AP_i_* and *AN_i_*	The existing contour, potential and new measures for *z_i_*, respectively.
*ACR_i_*, *APR_i_* and *ANR_i_*	The ranks of *z_i_* sorted by *AC_i_*, *AP_i_* and *AN_i_*, respectively.
Δ*AP_i_*, *әAP_i_*, Δ*AC_i_* and *әAC_i_*	The absolute and relative differences between *AP_i_* and *AN_i_* as well as between *AC_i_* and *AN_i_*.
Δ*APR_i_* and Δ*ACR_i_*	The ranking differences between *APR_i_* and *ANR_i_* as well as between *ACR_i_* and *ANR_i_*.
*LowZone_AC_*, *LowZone_AP_* and *LowZone_AN_*	The zones with the lowest ranks of *ACR_i_*, *APR_i_* and *ANR_i_*, respectively.
